# Defining window-boundaries for genomic analyses using smoothing spline techniques

**DOI:** 10.1186/s12711-015-0105-9

**Published:** 2015-04-17

**Authors:** Timothy M Beissinger, Guilherme JM Rosa, Shawn M Kaeppler, Daniel Gianola, Natalia de Leon

**Affiliations:** Department of Plant Sciences, University of California, Davis, 95616 USA; Department of Animal Sciences, University of Wisconsin, Madison, 53706 USA; Department of Biostatistics and Medical Informatics, University of Wisconsin, Madison, 53792 USA; Department of Agronomy, University of Wisconsin, Madison, 53706 USA; Department of Energy Great Lakes Bioenergy Research Center, University of Wisconsin, Madison, 53706 USA; Department of Dairy Science, University of Wisconsin, Madison, 53706 USA

## Abstract

**Background:**

High-density genomic data is often analyzed by combining information over windows of adjacent markers. Interpretation of data grouped in windows versus at individual locations may increase statistical power, simplify computation, reduce sampling noise, and reduce the total number of tests performed. However, use of adjacent marker information can result in over- or under-smoothing, undesirable window boundary specifications, or highly correlated test statistics. We introduce a method for defining windows based on statistically guided breakpoints in the data, as a foundation for the analysis of multiple adjacent data points. This method involves first fitting a cubic smoothing spline to the data and then identifying the inflection points of the fitted spline, which serve as the boundaries of adjacent windows. This technique does not require prior knowledge of linkage disequilibrium, and therefore can be applied to data collected from individual or pooled sequencing experiments. Moreover, in contrast to existing methods, an arbitrary choice of window size is not necessary, since these are determined empirically and allowed to vary along the genome.

**Results:**

Simulations applying this method were performed to identify selection signatures from pooled sequencing *F*_*ST*_ data, for which allele frequencies were estimated from a pool of individuals. The relative ratio of true to false positives was twice that generated by existing techniques. A comparison of the approach to a previous study that involved pooled sequencing *F*_*ST*_ data from maize suggested that outlying windows were more clearly separated from their neighbors than when using a standard sliding window approach.

**Conclusions:**

We have developed a novel technique to identify window boundaries for subsequent analysis protocols. When applied to selection studies based on *F*_*ST*_ data, this method provides a high discovery rate and minimizes false positives. The method is implemented in the R package GenWin, which is publicly available from CRAN.

## Background

A recurrent question that arises during the analysis of high-density genotyping or sequencing information is how to best analyze noisy data. This question is particularly relevant when analyzing sequence data from pooled samples of populations for which, depending on the number of individuals pooled and the level of coverage per site, estimates of individual base pair (bp) allele frequencies can be very imprecise [1]. To account for this variability, methods based on estimating parameters over “windows” have been successfully used to reduce sampling error while retaining true signal in studies aimed at identifying evidence of selection in populations [2-5]. In general, window-based techniques treat observations from individual genetic markers, often single nucleotide polymorphisms (SNPs), as samples that are representative of a phenomenon that affects isolated regions of the genome instead of independent SNPs. In studies aiming at identifying selection signatures, genetic hitchhiking [6] makes this approximation quite reasonable. It is also useful for other applications since, with the availability of increasingly denser marker arrays, linkage disequilibrium (LD) between SNPs within any particular region is likely to be substantial. Therefore, a summary statistic can be computed across a region or a window, instead of for individual SNPs. This summary statistic can be as simple as taking the mean of single-SNP estimates [3] or it can take a more complex form such as an aggregated measurement of divergence according to the Fisher’s angular transformation [4,7]. By using a sample of observations that are each considered as an estimate of the same phenomenon, as opposed to treating observations individually, sampling error may be markedly reduced, while retaining true signal. An inherent assumption of these methods is that the individual marker estimates within a window are independently and identically distributed.

Two types of approaches for delineating window boundaries are commonly used. These are referred to as “distinct windows”, for which markers in different windows do not overlap, and “sliding windows”, for which they do. When using distinct windows, the genome is divided into separate segments of equal length, with the length defined according to either the number of SNPs [4,8], or the number of base pairs (bp) [9]. A summary statistic that captures genomic patterns across each window, such as the mean *F*_*ST*_, is then calculated over all SNPs within a defined window. Distinct windows often succeed at reducing the sampling error of estimates while reducing the number of statistical tests performed, but the placement of windows is random or sequential, so power may be lost if window placement inadvertently splits one meaningful region into adjacent windows. In a sliding window approach, a window length (again in number of bp or SNPs) is defined and windows are incrementally advanced along the genome, a single or a few SNPs at a time, to ensure that every possible window is considered [5,10,11]. However, when using such an approach, the number of tests is not dramatically reduced since a new window is defined for every SNP or every few SNPs. In addition, highly correlated statistics are generated, since each window overlaps with its neighboring windows.

Beyond the limitations mentioned above, in the case of either distinct or sliding windows, determining the proper window size is typically subjective and researchers often only loosely justify their choice of size [10,12], or acknowledge that their choice is arbitrary [2,13]. This is unsatisfying for two reasons. First, there should be an optimum window size that balances noise reduction with signal identification to maximize power, and identifying this optimum would be ideal. Second, a subjective definition of window size typically leads to the use of a uniform window size across the genome, which is not appropriate since various genetic parameters, including recombination rate and LD, vary along each chromosome.

To address these problems, we have developed an empirically driven framework to define window boundaries, while simultaneously determining their ideal size. Our method retains the benefits of distinct windows in that it reduces the total number of tests and generates window values that are not inherently correlated, while also borrowing from sliding-windows by reducing the risk of erroneously dividing signal between adjacent windows. In addition, the ideal window size is automatically chosen and allowed to vary along the genome. The method is based on first fitting a cubic smoothing spline [14] to single-SNP estimates of a parameter such as *F*_*ST*_ [15]. Previously, various forms of smoothing splines have been used to analyze genomic information [16,17], but not to define windows. The smoothness of the spline is chosen by leave-one-out cross-validation, to ensure that it optimally predicts single-SNP values. The second derivative of the spline is then computed and inflection points are identified. The inflection points of the fitted spline isolate the positions where the spline switches from tending towards a local maximum to a minimum, or vice versa, and therefore DNA between these positions may correspond to a correlated region of the genome. Therefore, inflection points are treated as window boundaries and a distinct-window analysis proceeds. Using inflection points to define window boundaries virtually ensures that any peak in the fitted spline is placed in a single window instead of split across windows. Determining the fitted spline’s smoothness by cross-validation leads to ideal window-sizes. Moreover, although a uniform smoothness is chosen for the fitted spline, this does not explicitly restrict the location of its inflection points, thereby allowing non-uniformity of window sizes.

In this paper, we describe a smoothing spline-based approach to define windows using genomic data. In addition, we apply the method to both simulated and real data to identify signatures of selection, and demonstrate its advantages over previously used techniques. Although we present this method in the context of *F*_*ST*_-based studies, it can be applied to several other approaches that require the pooling of genotypic data over windows. This method has been implemented in a freely available R package, GenWin.

## Methods

### Spline technique

Noisy estimates of specific parameters such as *F*_*ST*_ can be obtained based on a series of individual markers along a chromosome. Therefore, observations from individual markers may be treated as estimates of an underlying continuous function *f* that specifies the true value of the statistic of interest at every position. Within this framework, various smoothing spline methodologies [14] may be used to estimate *f* and therefore its value at any position, *f(t*_*i*_*)*, where *t*_*i*_ is the chromosomal position in bp of marker *i*. If *f* is assumed to be continuous and twice differentiable, it may be approximated via a cubic smoothing spline [18]. The cubic smoothing spline estimate, $\widehat{f}$, of a function *f* applied over the range x*,* is defined as the solution that minimizes *S(f)*, where:

$$ S(f) = {\displaystyle \sum }{\left\{{Y}_i-f\left({t}_i\right)\right\}}^2+\lambda {\displaystyle \int }{f}^{\hbox{'}\hbox{'}}{(x)}^2dx\ . $$

Here, *Y*_*i*_ is the observed realization of the function and $\widehat{f}$ is restricted to be a member of the class of twice-differentiable functions. This formulation seeks to minimize the sum of squared errors of estimates obtained using $\widehat{f}$, while ensuring that $\widehat{f}$ is fairly smooth. This is achieved by penalizing the sum of squared errors by the integral of the squared derivative of *f*, at a rate determined by a smoothing parameter, λ [18]. This parameter may be chosen by cross-validation, so that the minimizer of *S(f)* is the function that provides the best predictive ability of the observed data. It has been shown [19] that $\widehat{f}$ is a piecewise-cubic polynomial, for which pieces are joined at marker positions and that even at these positions the first and second derivatives of $\widehat{f}$ are continuous.

**Figure 1:**
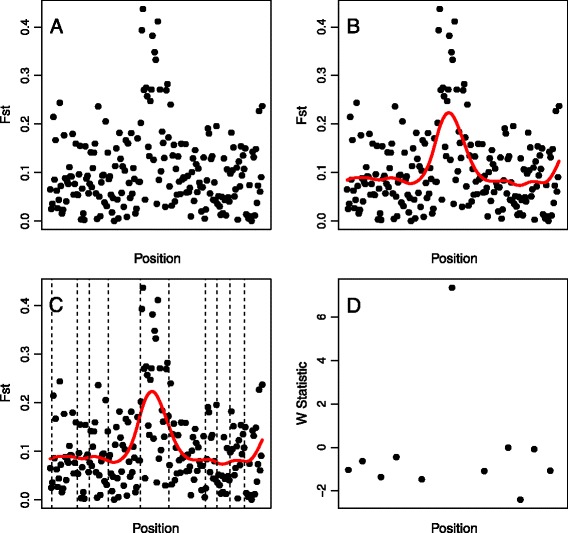
**Depiction of the method.** The spline-window method is presented step by step using a simulated set of 200 markers across a chromosome region. **(A)** Raw data (*F*_*ST*_) computed from individual markers. **(B)** A cubic smoothing spline indicated by the red line, is fitted to the data. **(C)** Inflection points of the spline are indicated by dashed vertical lines. **(D)** Inflection points of the spline are used to define window boundaries, and a statistic such as *W* is computed.

An overview of the smoothing spline method to define windows is provided in Figure 1. In the first step, a smoothing spline, $\widehat{f}$, is fitted to the raw, or unsmoothed, data, measured for individual SNPs. Next, this fitted spline is used to identify the positions at which the data are split for window-based analysis. Specifically, the inflection points of the fitted spline (positions where ${\widehat{f}}^{\hbox{'}\hbox{'}}=0$) are taken as window boundaries. Because $\widehat{f}$ will necessarily be concave-down at every local maximum, every potential peak in the spline and therefore every predicted peak in the underlying data are nearly ensured to fall into a single window, rather than being split between windows. In addition, large windows are created in regions where $\widehat{f}$ is mostly flat, which implies a low amount of signal relative to noise, and small windows are created in regions where $\widehat{f}$ is rougher, which indicates higher signal relative to noise. Once windows have been defined, analyses may proceed as with any methodology that involves genomic windows. However, the non-uniformity of window sizes must be appropriately accounted for in such analyses. Certain statistics naturally account for this variability. For instance, a simple *t*-test to assess changes in expected heterozygosity between two populations will appropriately handle differences in the number of observations per window. However, certain situations require a more cautious treatment of the variability in window sizes. *F*_*ST*_*-*based scans for selection, for example, often use outlier-based thresholds to identify potentially interesting regions with high *F*_*ST*_ values [12]. In this setting, seemingly high values from small windows may be less informative than seemingly intermediate values from large windows, due to the greater sampling error associated with fewer markers being included in smaller windows. A reasonable approximation is to consider individual markers as independent and identically distributed observations with some underlying mean value across the window. This assumption requires random variability about the window mean, which is likely to be achieved for reasonably small windows, and is no more of an assumption than is typically made for window-based analyses. Then, a *t*-test like statistic, *W,* may be generated such that:

$$ W = \frac{\left(\overline{X}-\mu \right)}{\sqrt{\raisebox{1ex}{${s}^2$}\!\left/ \!\raisebox{-1ex}{$n$}\right.}}, $$

where $\overline{X}$ is the mean value over the window, *μ* is the mean value over the entire dataset, *s*^2^ is the sample variance of *F*_*ST*_ across the entire dataset, and *n* is the number of observations (i.e. markers) in the window. Thus, each window will have a specific value of *W*, which may be used to compare among windows of varying size and to identify outliers. Similar to a *t*-test statistic, the *W* statistic penalizes the mean across each window according to its difference from the grand mean, the overall variability of the data, and most importantly, the number of individual observations used to compute that mean. Moreover, although *W* follows the form of a t-statistic, it is not expected to follow a t distribution, e.g., for selection scans where multiple generations of genetic drift add variability between populations. Still, *W* scales the means computed from windows of unequal size, so that comparisons are possible and outliers may be identified.

### R package

The smoothing spline method described above is implemented in the R [20] package GenWin. A cubic smoothing spline is fitted to single-SNP estimates of some parameter of interest, e.g. *F*_*ST*_, accounting for the position of each estimate in bp. These single-SNP estimates should be calculated externally and provided to GenWin by the user. GenWin depends on the “pspline” package [21] for a rapid fitting of a cubic smoothing spline to data. The smoothing parameter may be chosen within GenWin via cross-validation (CV) or generalized cross-validation (GCV) [22]. The inflection points of the spline are identified as the points for which the second derivative switches sign, and the user may specify the resolution over which second derivatives are computed. Computing second derivatives at every bp slows down computation and may lead to erratic window boundaries, but doing this only every few thousand bp, may allow properties of the fitted spline to be missed. By default, GenWin looks for inflection points at a resolution of 100 bp. Users that desire finer isolation of inflection points may modify the “smoothness” parameter to a smaller value, although informal testing suggests that a 100 bp resolution is adequate. After inflection points are identified, these are used to define window boundaries, and a variety of statistics for each window, including means and the *W* statistic described above, are returned. Plotting the spline fitted to raw data and plotting values of the *W* statistic are optional. Only one chromosome should be analyzed at a time, since the generation of a function that is continuous between the end of one chromosome and the beginning of another is not biologically meaningful. The splineAnalyze() function takes between a few seconds and a few minutes on a typical workstation to analyze 100 000 markers that may lead to several thousand windows, depending on the smoothness of the fitted spline.

### Simulations

The software QMSIM [23] was used to simulate an artificially selected population that was suited for testing the spline-window method. A diploid species with 10 chromosomes, each chromosome being 200 centiMorgans (cM) and 100 Mb long, was simulated. First, 5000 historical generations with 5000 random mating individuals per generation were simulated to establish a base population for selection. No selection took place during the 5000 historical generations. Next, 100 replications of selection were carried out on a trait that was controlled by 30 quantitative trait loci (QTL) and had a heritability of 0.5. Selection based on “high” phenotypes was carried out for 30 discrete generations, with 500 males and 500 females selected to contribute gametes at each generation, and a litter size of 50 individuals per female (i.e. census population size = 25 000). Three QTL and 100 000 markers were simulated on each chromosome. Marker positions were assigned randomly, and on each chromosome, three QTL were placed at precisely 50, 100 and 150 cM. QTL effects were randomly sampled from a normal distribution. Markers and QTL were both di-allelic, and recurrent mutation was permitted during the historical generations at a rate of 2.5 × 10^-5^.

The output from QMSIM included allele frequencies for each of the 1 000 000 markers pre- and post-selection, in each of the 100 replicated populations. Binomial sampling was conducted within R (R Core Team, 2013) to further simulate a set of pooled genotyping data for analysis. For every marker within each simulated replicate, in each of the pre-selection and post-selection populations, 100 individuals (200 gametes) were sampled to create a simulated set of individuals for genotyping, and then 50 binomial samples were drawn from those individuals to approximate pooled sequencing to 50X coverage. It has been shown that pooled sequencing is well approximated by binomial sampling [12]. Thus, this process generated a set of estimated allele frequencies corresponding to a population that had undergone 30 generations of selection, then 100 individuals were sampled and sequenced, with a sequencing depth equivalent to a 50X coverage.

In general, simulations such as the one performed here are analyzed using outlier-thresholds to identify potentially selected sites, e.g. [24]. However, since this population was fully derived via simulation and all parameters were known, we were able to simulate the population without selection to define significance thresholds. For this model, the only changes to the previously described protocol were that individuals were selected independently of their performance for the given trait, and 20 replicated populations (again including 1 000 000 di-allelic markers per population) were simulated.

To evaluate the spline-window method’s performance compared to either sliding or distinct windows of various sizes, *F*_*ST*_ values between the pre- and post-selection populations were computed for each marker according to ${F}_{ST} = \frac{s^2}{\overline{p}\left(1-\overline{p}\right)+{s}^2/r}$, where *s*^2^ is the sample variance of allele frequency between populations, $\overline{p}$ is the mean allele frequency across populations, and *r* = 2 is the number of populations [15]. Sliding window and distinct window values were computed for windows of five, 10, 25, 50, 100, 250, and 500 SNPs. In addition, the spline-window method was also tested using the *W* statistics to compare between windows of unequal size with GCV and a resolution of 100 bp. Significance thresholds at the multiple-testing-corrected 5% significance level for each method were determined via simulations without selection. To achieve this, the maximum observed value in each unselected replication was identified, and the 95% quantile of these values was taken as the threshold. Finally, the simulations were analyzed for true positive (i.e. detection) and false positive rates. Windows that exceeded the simulated significance thresholds were deemed true positives if they fell within 5 cM of a simulated QTL, and were deemed false positives otherwise. 5 cM is a conservative distance due to the fact that selection affects a segment of DNA. Thus, depending on parameters such as population size and recombination rate, it is expected that selection will affect patterns of variability surrounding the causative polymorphism. In other words, treating all windows that do not contain the precise location of a QTL as false positives would lead to an excessive number of false positives for all methods.

### Empirical data analysis

We re-analyzed a maize dataset that was previously published [5] and that involved a population subjected to artificial selection for 30 generations to increase the number of ears per plant. Pooled sequencing was conducted pre- and post-selection, and estimated *F*_*ST*_ values computed between pre- and post- selection populations at approximately 1.2 million SNPs were available. In the previous analysis, sliding windows of 25 SNPs were used, and a 99.9% outlier threshold was applied to identify the most divergent regions of the genome that were likely to have been under selection pressure. For this re-analysis, the spline-window method was applied using GCV to choose the smoothing parameter. Again, a 99.9% outlier threshold was applied to identify outlying *W* statistics that identify regions with a likely selection signature. For consistency with the previous analysis, outlying windows within 5 Mb of one another were grouped together since they probably correspond to the same selection event. Results from the spline-window analysis were compared to those of the previously published sliding-window analysis to determine the degree of overlap between the methods.

## Results

### Simulations

Simulations showed that both sliding windows and distinct windows of five or 10 SNPs identified markedly fewer QTL than larger window sizes (Table 1). With such small windows, data varied greatly and therefore the significance thresholds that were set in the simulations without selection were so high that it was extremely difficult to exceed them. The positive aspect of this, however, is that these four methods identified fewer false positives than observed with larger window sizes. In fact, the distinct window methods with five or 10 SNPs per window identified the fewest false positives of all methods investigated. For these methods, however, low false positive rates came at the expense of high detection rates. For instance, either sliding or distinct windows of only five SNPs identified, on average, fewer than 25% of the simulated QTL, which was substantially less than the other methods. Conversely, all sliding and distinct window implementations of 25 or more SNPs, as well as the spline-window method, identified a similar number of QTL on average, all with mean detection rates greater than 50% of the total number of QTL but less than 66.67%. It should be noted that, depending on the allele frequencies and effect sizes of QTL at the beginning of selection, it is not expected that every QTL will be detectable, so maximum detection rates lower than 66.67% are not necessarily surprising. Between methods, the number of false positives that were identified varied greatly, with the sliding window methods showing the greatest number of false positives. For this reason, the ratio of detected QTL to false positives was used to evaluate the performance of each method. Excluding the 5- or 10-SNP window methods due to their low detection rates, the ratio of detection rate to false positive rate of the spline-window method (4.7) was more than double that of the second best-performing method, which used distinct windows of 25 SNPs (2.2).

**Table 1:** **Method comparison using simulated data**

**Method**	**Mean number of detected QTL**	**Mean number of false positives**	**Ratio**
**Sliding-5**	6.33	6.39	0.990610329
**Sliding-10**	13.29	4.16	3.194711538
**Sliding-25**	17.6	75.1	0.234354194
**Sliding-50**	18.38	232.58	0.079026572
**Sliding-100**	18.23	488.19	0.037342018
**Sliding-250**	18.41	2082.54	0.008840166
**Sliding-500**	19.51	9065.05	0.002152222
**Distinct-5**	7.42	0.12	61.83333333
**Distinct-10**	12.19	0.99	12.31313131
**Distinct-25**	16.75	7.67	2.183833116
**Distinct-50**	18.01	11.27	1.598047915
**Distinct-100**	17.73	9.52	1.862394958
**Distinct-250**	19.49	31.53	0.618141453
**Distinct-500**	18.34	28.47	0.644186863
**Spline Windows**	15.98	3.4	4.7

Results from applying an assortment of window-methods applied to 100 simulated selection experiments involving 30 QTL, 30 generations of selection, and pooled sequencing at 1 000 000 markers to estimate allele frequencies. The mean number of QTL (out of 30) detected over the 100 simulations, mean number of false positives, and ratio of detections to false positives across simulations is provided for each of the methods evaluated. Sliding- and Distinct- refer to sliding and distinct window methods with windows of the specified size, and Spline Windows refers to the method described here and employed in GenWin, where window size is not restricted a priori.

Two important remarks should be made. First, of all the methods, the ones that used 5- or 10-SNP distinct windows had the most favorable ratio of detection rate to false-positive rate (61.8 and 12.3, respectively), but this was at the cost of identifying notably fewer QTL. Therefore, if the aim is to identify only the most promising sites, a suitable approach would be to adopt a sliding or distinct window method with a relatively small window size and take only the most outlying windows for further study. This approach is likely to find extreme QTL, and by limiting the search to only a few of these, the expectation is that the number of false positives will be small. The second remark is that, for any method, the ideal window size is determined by a complex interplay between the true signal in the data and the amount of error that results from sampling and genotyping. Therefore, there is no single ‘ideal’ window size that will hold across experiments but, instead, the best window size will vary depending on the genetic structure underlying the trait under study and the genotyping methods applied. The spline-window method provides a useful alternative by letting the variability in the data determine the appropriate window size.

### Real data analysis

**Figure 2:**
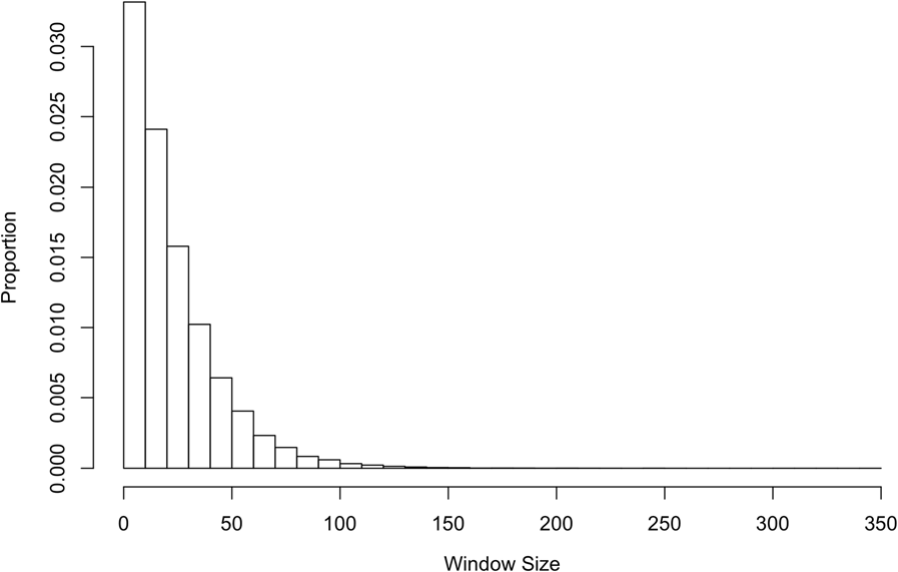
**Window sizes.** Histogram of the variability in window sizes, shown according to number of markers included per window, obtained by applying the spline window method to previously published maize data [5].

The spline-window analysis of the previously published maize data identified 23 unique regions that exceeded the 99.9% empirical outlier level and which were expected to be associated with selection (Table 2). Within these 23 regions, 17 overlapped with those identified in the previously published analysis [5], while six were novel regions that had not been identified using sliding windows (in one case two spline-based regions corresponded to a single previously reported region). In addition, 12 of the regions that had been identified in the previous study [5] were no longer outliers according to the spline-window analysis. As expected, a substantial amount of variability in the size of windows was observed using the spline method. While the previous study restricted the size of all windows to precisely 25 SNPs, the spline approach suggested that approximately 64.3% of windows should have less than 25 and 34.0% should have more than 25 SNPs, with only 1.6% of windows having exactly 25 SNPs (Figure 2). Moreover, 10% of the windows had more than 51 SNPs and the maximum fitted window size was 349 SNPs, which implies that a large amount of variability in the noisiness of the data will be inappropriately accounted for if a single window size is used.

**Table 2:** **Application of sliding window and spline methods to empirical data**

	**25-SNP sliding window outlier regions**		**Spline-window outlier regions**	
**Chromosome**	**Start position**	**End position**	**Start position**	**End position**
1	11 588 371	11 892 655	11686850	11872650
1	-	-	54485850	54564950
1	122 802 601	122 831 005	122 790 650	124 093 750
1	164 947 151	165 229 053	-	-
2	35 519 192	35 682 346	35 520 750	35 648 950
2	41 731 365	41 755 299	41 728 850	41 770 550
2	71 306 928	71 378 431	71 314 050	71 377 150
2	101 062 088	101 069 759	101 037 150	102 026 750
2	160 786 800	160 802 631	-	-
3	177 548 249	177 681 538	177 671 050	177 749 050
3	-	-	207 464 650	211 847 850
3	215 594 013	215 778 968	-	-
4	66 924 240	66 935 990	-	-
4	82 825 221	82 858 997	82 818 050	82 860 750
4	113 455 144	122 680 452	113 401 750	114 347 650
			120 298 350	122 682 750
4	-	-	140 791 850	140 834 650
4	191 396 139	191 400 390	-	-
5	-	-	24 460 850	24 539 450
5	30 083 952	30 139 317	30 034 650	30 120 950
6	41 490 195	45 914 266	41 517 550	45 921 450
6	75 749 792	76 382 768	76 072 450	76 176 350
6	-	-	86 671 650	86 727 750
6	119 682 711	119 692 810	119 683 750	119 707 650
7	146 671 419	146 771 150	-	-
7	167 742 364	167 809 449	-	-
8	92 876 772	94 647 137	94 633 950	94 680 950
8	118 681 864	118 767 444	-	-
9	26 149 935	26 181 104	25 947 850	26 183 950
9	101 071 793	101 097 690	-	-
10	7 635 223	8 719 903	8 703 450	8 718 950
10	18 846 988	19 024 881	-	-
10	25 251 913	25 264 660	-	-
10	97 503 134	97 542 318	-	-
10	-	-	136 171 150	136 259 150

A comparison of regions exceeding a 99.9% threshold using 25-SNP sliding windows and spline windows, based on empirical data. The data analyzed are from [5], a study on a 30-generation artificial selection experiment for maize ear number. Previously published outlying regions identified as putatively controlling number of ears by plant based on 25-SNP sliding windows are compared with those identified applying the spline-window method to the same dataset.

**Figure 3:**
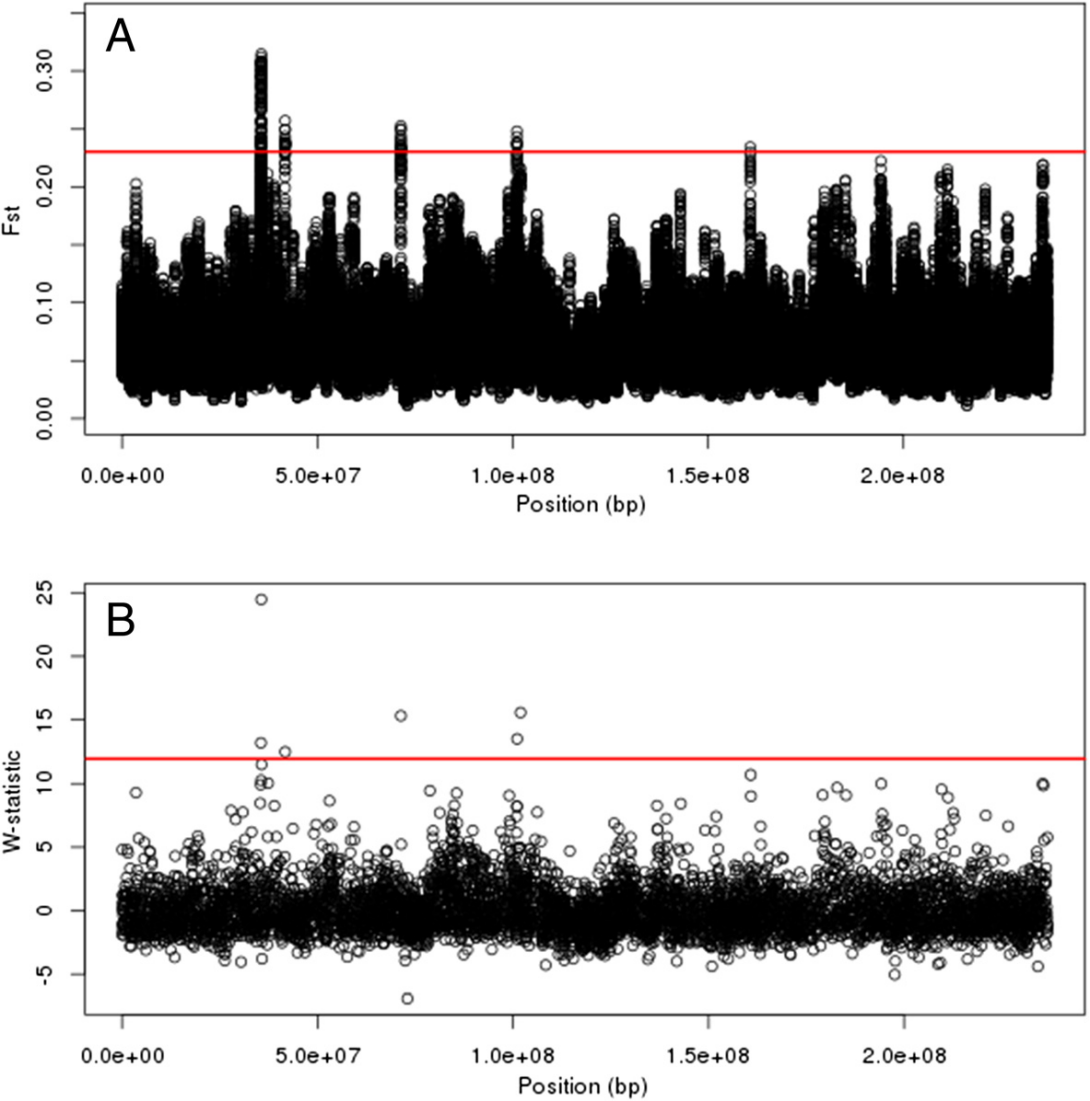
**Comparison to previously published data.** A comparison of regions exceeding a 99.9% threshold using 25-SNP sliding windows and spline windows, based on empirical data. The data analyzed are from a previous study on a 30-generation artificial selection experiment for maize ear number [5]. **(A)**: Adapted from [5], *F*_*ST*_ values and their outlier threshold (red line) found on maize chromosome 2 using 25-SNP sliding windows. **(B)**: *W* statistics and their outlier threshold (red line) found using the spline-window method for the same dataset.

In addition, the spline-window approach appeared to be superior at separating outlying regions from the background variability of the data compared to the previous analysis that used 25-SNP sliding windows. While sliding windows necessarily lead to correlations between adjacent windows, causing an outlying window to be surrounded by other windows that are also outlying or nearly outlying, spline-based windows do not share this property. Specifically, defining windows based on smoothing splines allows each significant or outlying region to have a clearly defined start and stop position at the underlying inflection points of the spline. Therefore, except in cases of selective-sweeps that spanned several Mb, outliers identified based on the inflection points of the fitted spline were generally well distinguished from their neighbors (Figure 3).

## Discussion

This study demonstrates that arbitrarily defining a window size for the analysis of high-throughput genotypic data and then proceeding to analyze an experiment across sliding or distinct windows of the specified size has some limitations. Small window sizes tend to decrease the potential discovery rate of the study, while large window sizes tend to create an abundance of false positives. The spline-window method avoids both extremes by using patterns in the data to define windows. The windows are placed at potential peaks, and their size is determined by the variability present in the data.

The results of the simulation analysis established that the spline-window method achieved a balance between discovery rate and false positive rate that was considerably better than any of the other methods that were examined and identified a comparable number of QTL. Our re-analysis of previously published data demonstrated that this technique performs well in experimental situations. The simulated scenario used here is expected to represent the biology of the previously published data [5]. Based on that assumption, the results suggest that the detection rates of the spline-window and 25-SNP sliding window methods should be similar, while the false positive rate of the sliding window method is substantially higher than that of the spline-window approach. This is consistent with the identification of 23 selected regions with the spline-window method and of 28 regions in the previously published data [5], of which 17 overlapped.

The spline-window method has potential to be used across multiple types of studies, although in this study, it was used in the context of *F*_*ST*_-based scans for selection signatures. This method may be applicable to various situations where noisy genomic data are divided into windows for analysis. For example, the d_i_ statistic [25], evaluations of heterozygosity, e.g. [2], and similar metrics that can be computed for individual loci or across windows fit into this framework very well, since a smoothing spline can be fitted to single-locus estimates of any statistic before window-based smoothing is performed. Extending this approach to statistics such as Tajima’s D [26] or extended haplotype homozygosity [27] and variants thereof may be possible as well, but this is not straightforward since these statistics are computed across windows in the first place, and therefore the values that the spline should be fitted to are not as clear.

There are some areas of study that may prove fruitful to improve and extend this method. The first involves the smoothing parameter, λ. When this parameter is chosen via cross-validation, a single value is used for each chromosome. Since recombination rates, and therefore levels of genomic variability, can vary substantially along a chromosome, this approach may benefit from an extension to include multiple smoothing parameters that are fitted simultaneously to different regions within a chromosome. For example, an ideal spline may be smoother (resulting from a larger smoothing parameter) in centromeric and peri-centromeric regions than elsewhere. Secondly, it is difficult to adequately estimate LD from pooled sequencing data. There has been progress towards this goal, as described in [28], but the short length of the sequencing reads that are currently obtained, relative to typical distances of LD decay, represents a substantial limitation. Spline-windows are likely determined by underlying levels of LD and recombination, and therefore there may be a possibility to extend this general approach as a means to assess LD in pooled sequencing situations.

## Conclusions

An important component of the analysis of data in studies that involve high-density genomic sequence information is how to best group regions of the genome for analysis. This is particularly relevant to identify selection signatures based on pooled sequencing data, for which estimates of features, such as *F*_*ST*_, contain substantial sampling error. We proposed a spline-based method that simultaneously defines ideal boundaries and variable sizes for windows of observations that may be analyzed together. Simulations coupled with empirical data analysis demonstrated that the power of this method is similar to that of existing methods but that it is less susceptible to false positives. We have made this method freely and publicly available in the R package GenWin.
